# Association of milk microbiome with bovine mastitis before and after antibiotic therapy

**DOI:** 10.14202/vetworld.2023.2389-2402

**Published:** 2023-12-05

**Authors:** Inna Burakova, Mariya Gryaznova, Yuliya Smirnova, Polina Morozova, Vitaliy Mikhalev, Vitaliy Zimnikov, Irina Latsigina, Sergey Shabunin, Evgeny Mikhailov, Mikhail Syromyatnikov

**Affiliations:** 1 Laboratory of Metagenomics and Food Biotechnology, Voronezh State University of Engineering Technologies, 394036 Voronezh, Russia; 2 Department of Genetics, Cytology and Bioengineering, Voronezh State University, 394018 Voronezh, Russia; 3 FSBSI All-Russian Veterinary Research Institute of Pathology, Pharmacology and Therapy, 394061 Voronezh, Russia.

**Keywords:** cattle, dairy industry, early diagnosis, microbiome

## Abstract

**Background and Aim:**

Mastitis is recognized as the most common disease in cattle and causes economic losses in the dairy industry. A number of opportunistic bacterial taxa have been identified as causative agents for this disease. Conventionally, antibiotics are used to treat mastitis; however, most bacteria are resistant to the majority of antibiotics. This study aimed to use molecular methods to identify milk microbiome patterns characteristic of mastitis that can help in the early diagnosis of this disease and in the development of new treatment strategies.

**Materials and Methods:**

To evaluate the microbiome composition, we performed NGS sequencing of the *16S rRNA* gene of the V3 region.

**Results:**

An increase in the abundance of the bacterial genera *Hymenobacter* and *Lachnospiraceae NK4A136 group* is associated with the development of subclinical and clinical mastitis in dairy cows. These bacteria can be added to the list of markers used to detect mastitis in cows. Furthermore, a decrease in the abundance of *Ralstonia*, *Lachnospiraceae NK3A20 group*, *Acetitomaculum*, *Massilia*, and *Atopostipes* in cows with mastitis may indicate their role in maintaining a healthy milk microbiome. Antibiotics reduced the levels of *Streptococcus* in milk compared to those in the healthy group and cows before antibiotic treatment. Antibiotic therapy also contributed to an increase in the abundance of beneficial bacteria of the genus *Asticcacaulis*.

**Conclusion:**

This study expands our understanding of the association between milk microbiota and mastitis.

## Introduction

Mastitis is a major problem in dairy farming because of its broad detrimental effects on cattle and humans. It is a polybacterial disease and is being investigated throughout the world [1]. Mastitis in cattle is inflammation of the parenchyma tissue of the udder, which can result in physical, chemical, and bacteriological changes in the milk and pathological changes in the glandular tissue [2]. Symptomatically, mastitis can be divided into clinical and subclinical [3, 4], of which subclinical mastitis is the most common, leading to decreased milk production without visible clinical signs. Therefore, it is difficult to diagnose this disease, and it persists longer in the herd [5]. The major mastitis-causing pathogens are *Streptococcus uberis*, *Streptococcus* *dysgalactiae*, *Staphylococcus* *aureus*, and *Escherichia coli* [6]. These pathogens are transmitted through various modes such as the hands of farmworkers and hygiene equipment for washing udders.

The primary treatment for mastitis generally involves intrathoracic infusion or parenteral antibiotics such as streptomycin, ampicillin, cloxacillin, penicillin, and tetracycline [7]. However, bacteria isolated from the milk of cows with mastitis often exhibit antibiotic resistance, with the maximum antimicrobial resistance observed against benzylpenicillin (56.3%) and oxytetracycline (46.2%) [8]. The effectiveness of mastitis treatment in cattle depends on the sensitivity of pathogens to antimicrobials, the type of mastitis, the breed of cattle, and the treatment regimen [9]. Emergence of drug resistance is a major challenge in mastitis control because antibiotic resistance profiles are often herd-specific [10]. Rapid identification and understanding the diversity of pathogens associated with mastitis are essential for effective disease prevention and control [11].

This study was conducted to investigate the microbiome in the milk samples of cattle with clinical and subclinical manifestations of mastitis, as well as healthy cattle, by sequencing the *16S rRNA* gene (V3 hypervariable region) on the Ion Torrent PGM platform (Madison, WI, USA). The microbial community of milk in groups of cows with mastitis after antibiotic treatment was also compared. This study would allow us to better evaluate the role of the milk microbiome in bovine mastitis and design further studies to collect information and develop an effective treatment strategy for bovine mastitis.

## Materials and Methods

### Ethical approval

The animal study protocol was approved by the Ethics Committee of FSBSI “All-Russian Veterinary Research Institute of Pathology, Pharmacology and Therapy” (protocol number 1-02/23, 10 February 2023).

### Study period and location

This study was conducted from February 2023 to May 2023 (90 days) in FSBSI All-Russian Veterinary Research Institute of Pathology, Pharmacology and Therapy and Laboratory of Metagenomics and Food Biotechnology of Voronezh State University of Engineering Technologies, Voronezh, Russia.

### Objects of study

Holstein cows, whose milk yield for the last lactation was 6870–7650 kg, were selected for this study. The animals were maintained under controlled conditions, with optimal room temperature (30 ± 2°C) and humidity (55% ± 7%). They were fed 3 times a day with a complete diet, which was calculated considering the indicators of average productivity. A total of 15 cows with various udder pathologies were selected and further divided into three groups. Table-1 shows the data on the study groups.

**Table-1:** Groups of the studied animals.

No. of sample	Clinical diagnosis
1-5	Cows with acute clinical mastitis
6-10	Cows with subclinical mastitis
11-15	Clinically healthy cows

The first group consisted of clinically healthy animals with 2–3 lactations that had not previously suffered from mastitis, the second group included cows with subclinical mastitis with 2–3 lactations, and the third group consisted of cows with acute clinical mastitis with 2–3 lactations. The group of clinically healthy cows included animals without clinical signs of mastitis, with a negative reaction to the Keno™ test (CID LINES, Ypres, Belgium), the number of somatic cells being <200,000/mL, and no history of diagnosis of “mastitis.” The exclusion criteria from the group of clinically healthy animals were an increase in the number of somatic cells to >200,000/mL, a positive reaction to the Keno™ test, and clinical signs of mastitis or other diseases. The group of cows with subclinical mastitis consisted of animals with a positive reaction to the Keno™ test during a 2-time study with an interval of 48 h, without clinical signs of mastitis, and the number of somatic cells being >1 million/mL. The exclusion criteria from the group of cows with subclinical mastitis were the number of somatic cells in the milk of <1 million/mL and the presence of clinical signs of mastitis and other diseases. The clinical mastitis group included cows in which one or two lobes were enlarged in volume, dense to the touch, and hyperemic. The udder secretion was watery and contained clots of casein. The exclusion criterion from the group of cows with clinically pronounced mastitis was the absence of clinical signs of mastitis or the presence of clinical signs of other diseases. Table-2 shows the details of clinical observations of the cows.

**Table-2:** Clinical observations of cows.

Indicators	Animal groups		

	Clinically Healthy	Subclinical mastitis	Acute clinical mastitis
Temperature, °C	37.7 ± 0.6	38.6 ± 1.4	39.7 ± 1.5
Pulse beats/min	75.6 ± 6.1	81.4 ± 2.8	82.6 ± 4.7
Breathing, respiratory, movements/min	14.4 ± 1.2	16.0 ± 1.3	16.8 ± 1.5
Reaction with Keno™ test	-/-	+++	not done
Sediment test	10–2.2 0.0	10–1.6 0.0	10–0.7 0.0
Somatic cells, thousand/mL	<200	>1000	>3600
Milk properties	Without changes	No visible changes	Watery with casein clots

*Keno™ test (CID LINES, Ypres, Belgium). Keno test is a test aimed at the rapid detection of subclinical mastitis. The principle of operation, which is based on the S.M.T. (California mastitis test), gives a semi-quantitative measure of the level of somatic cells present in milk

Bacteria of the genera *Streptococcus*, *Staphylococcus*, *Enterococcus*, and *Enterobacter* were isolated from the cows’ milk (Table-3). Animals from groups 2 and 3 received mastitis treatment via intracisternal administration of a broad-spectrum antimicrobial preparation, which included tetracycline hydrochloride, neomycin sulfate, and bacitracin, according to the prescription, 4 times twice a day with an interval of 12 h during the study period. The antibiotic drugs were selected based on testing the sensitivity of the isolated pathogens to antibiotics. Treatment of cows with subclinical and clinical mastitis was performed immediately after diagnosis. The indicators of cow udder secretion before and after mastitis treatment are shown in Table-4.

**Table-3:** Bacteria isolated from cow’s milk.

Group	Species composition of milk microflora	Occurrence, %
Clinically healthy	*Streptococcus disgalactiae*	31.9
	*Staphylococcus epidermidis*	11.2
	*Enterococcus faecium*	30.5
	*Enterococcus faecium*	26.4
Subclinical mastitis	*Streptococcus aureus*	28.7
	*Enterococcus faecalis*	37.5
	*Enterobacter cloacae*	33.8
Acute clinical mastitis	*Streptococcus agalactiae*	27.7
	*Staphylococcus aureus*	16.3
	*Enterococcus faecalis*	29.5
	*Enterococcus faecium*	26.5

**Table-4:** Indicators of cow udder secretion before and after mastitis treatment.

Indicators	Before treatment	After treatment
Clinically healthy		
Protein, %	3.12 ± 0.01	3.13 ± 0.03
Fat, %	3.82 ± 0.09	3.86 ± 0.06
Density, kg/m^3^	1029.4 ± 0.6	1029.3 ± 0.4
Somatic sells, thousand per mL	184.5 ± 30.6	174.4 ± 45.2
Milk productivity kg per day	24.5	24.7
Subclinical mastitis		
Protein, %	2.85 ± 0.20	3.16 ± 0.01
Fat, %	3.41 ± 0.10	3.83 ± 0.12
Density, kg/m^3^	1026.4 ± 0.5	1028.5 ± 0.2
Somatic sells, thousand per mL	1748.3 ± 259.3	287.0 ± 35.4
Milk productivity kg per day	20.3*	23.5
Acute clinical mastitis		
Protein, %	2.45 ± 0.08*	3.18 ± 0.01
Fat, %	3.21 ± 0.11*	3.74 ± 0.06
Density, kg/m^3^	1024.7 ± 0.6	1027.3 ± 0.3
Somatic sells, thousand per mL	2550.4 ± 162.4***	383.0 ± 22.3
Milk productivity kg per day	15.2***	19.3

* (р < 0.05);

*** (р < 0.001)

Milk samples were collected twice, namely, the first time before the start of treatment and the second time after the completion of the therapeutic course. Milk sampling was performed according to the guidelines for the bacteriological examination of milk and cow udder secretion (Guidelines 115-69, in the Russian Federation).

Milk was collected from the quarters of the udder in compliance with the rules of asepsis. Accordingly, the udder teats and hands of the staff were treated with a 70% alcohol solution before collecting a milk sample. Samples of alveolar milk, 5–10 mL each, were collected at the end of milking into sterile plastic tubes. During sampling, the staff ensured that the udder did not touch the edge of the tube. The tubes were then tightly closed with sterile caps. After milk sampling, the test tubes were immediately placed in liquid nitrogen for further transportation to the laboratory.

### Sequencing of the *16S rRNA* gene of the V3 region

DNA was extracted from the obtained milk samples using the commercial HiPure DNA Micro Kit (Magen, China), according to the manufacturer’s protocol.

Polymerase chain reaction (PCR) analysis of the hypervariable region V3 of the *16S rRNA* gene was conducted to further analyze the bacterial composition using the Ion Torrent PGM platform. A pair of universal primers were used for the reaction, viz., 337F: 5'-GACTCCTACGGGAGGCWGCAG-3'; 518R: 5'-GTATTACCGCGGCCTGCTGG-3', as well as the commercial mix 5X Screen Mix-HS Master MixKit (Evrogen, Russia). The amplification protocol consisted of the following steps: Total denaturation at 94°C for 4 min, 25 cycles of denaturation at 94°C for 30 s, primer annealing at 53°C for 30 s, elongation at 72°C for 30 s, and final elongation at 72°C for 5 min.

For the subsequent preparation of sequencing libraries, the PCR products were purified using AMPureXP magnetic particles (BeckmanCoulter, Brea, CA, USA), after which a commercial NEBNext Fast DNA kit (New England Biolabs, Ipswich, MA, USA) was used according to the manufacturer’s protocol. The libraries were then barcoded using a commercial NEXTflex Kit (Ion Torrent; 64 adapters; PerkinElmer, Inc., Waltham, MA, USA) and also purified using AMPureXP magnetic beads (Beckman Coulter, Brea, CA, USA).

Next, the libraries were enriched using emulsion PCR and the Ion PGM Hi-Q View sequencing kit for post-processing and loading on a chip (Thermo Fisher Scientific, Madison, Wisconsin, USA). For further sequencing, the libraries were loaded onto Ion 318™ Chip v2 BC using standard Ion PGM Hi-Q View OT2 kit protocols.

### Statistical analysis

Statistical processing of the extracted data was performed using the R programming language in the R Studio environment (V SEARCH v software.2.8.2; version 1.1.414 © RStudioInc., RStudio PBC, 2009-2018, Boston, Massachusetts, USA).

First, BAM files were developed for each sample, which were converted to the FastQ format based on the FileExporter plugin. Invalid reads were excluded using the maximum predicted error threshold, for each reading, whose value was <1.0 (DADA2 package). Moreover, all reads were optimized to a common size with concomitant demultiplexing. For subsequent analyses, dereplication was performed, through which all identical reads were combined into unique sequences. Next, operational taxonomic units were compiled based on the UNOISE2 algorithm.

Identification was performed using version 138 of the SILVA database (https://www.arb-silva.de/, accessed 15 April 2023). The taxonomy was typed relative to a peak similarity threshold, with variant amplicon sequences equivalent to 97%. All raw sequences extracted from the analysis of each sample are available from the BioProject repository (Bio Project: PRJNA862929).

Differential analysis of abundance was conducted using DESeq2, in which p values derived from the Wald test in pairwise testing were corrected using the Benjamini and Hochberg method. Alpha-diversity was evaluated using the Shannon index. The statistical significance of the observed differences was evaluated using a pairwise test with the Wilcoxon rank sum test, adjusted using the false discovery rate (FDR) method. Differences in microbiome composition in the examined milk groups were evaluated using non-metric multivariate scaling (NMDS) using Bray-Curtis dissimilarity consistent with the analysis of similarities test.

## Results

A total of 1,219,921 reads with an average length of 175 bp were obtained by sequencing on the next-generation semiconductor sequencer Ion Torrent PGM. All raw data were stored in the NCBI BioProject repository (BioProject: PRJNA736244). After qualitative filtration, 1,49,196 reads were used for further analysis, which corresponded to 264 bacterial genera.

**Figure-1:**
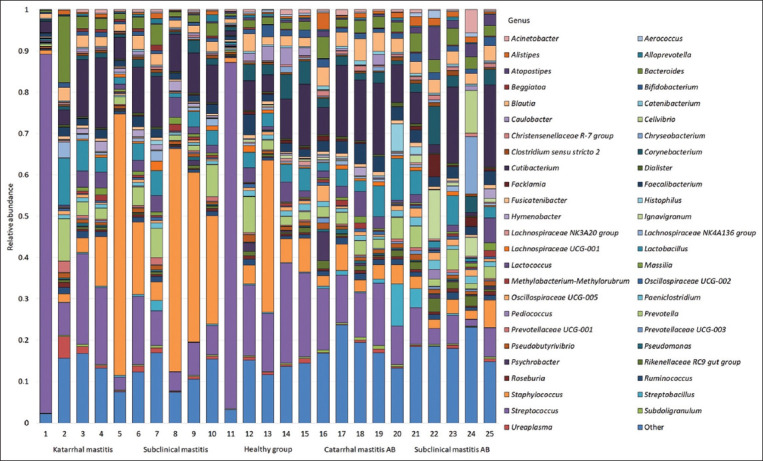
Abundance bars for the most common bacterial genera.

We identified the top bacteria, which included 49 bacterial genera, whose proportion for one of the study groups exceeded 0.5%, and the remaining representatives were combined into “Other” (Figure-1).

**Figure-2:**
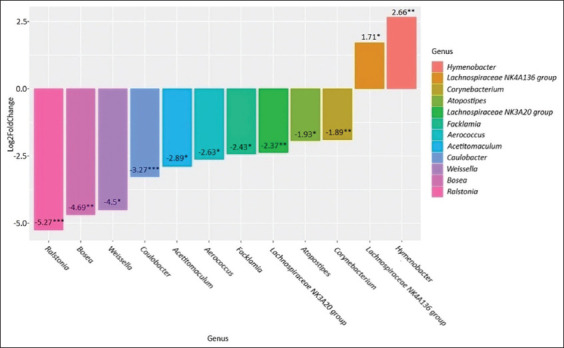
Genus-level differences in the clinical mastitis group compared to the healthy group. *p ≤ 0.05, **p ≤ 0.01, ***p ≤ 0.001.

**Figure-3:**
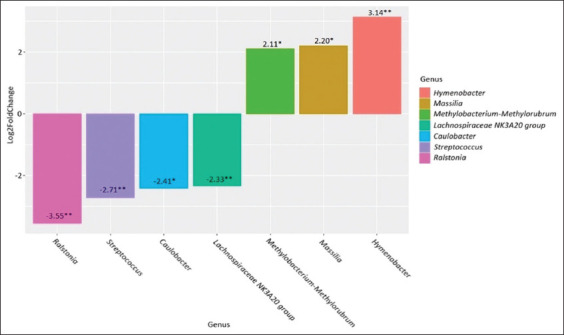
Genus-level differences in the subclinical mastitis group compared to the healthy group. *p ≤ 0.05, **p ≤ 0.01, ***p ≤ 0.001.

**Figure-4:**
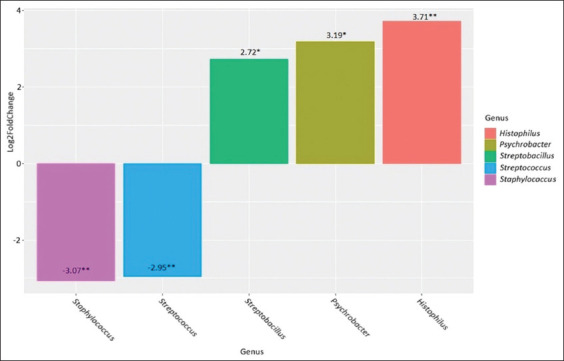
Genus-level differences in the clinical mastitis group after antibiotic therapy compared to the clinical mastitis group before treatment. *p ≤ 0.05, **p ≤ 0.01, ***p ≤ 0.001.

**Figure-5:**
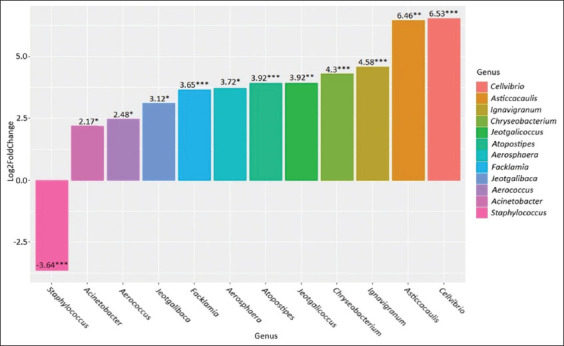
Genus-level differences in the subclinical mastitis group after antibiotic therapy compared to the subclinical mastitis group before treatment. *p ≤ 0.05, **p ≤ 0.01, ***p ≤ 0.001.

Our results showed that *Streptococcus* was predominant in cows with clinical mastitis and healthy cows (27.70% and 31.93%, respectively). In cows with subclinical mastitis, *Staphylococcus* was predominant (28.67%). *Staphylococcus* was also the second most abundant genus in the clinical mastitis group (16.34%) and the healthy group (11.23%). In both groups after antibiotic treatment, *Cutibacterium* was found to be dominant, with its abundance being 12.98% in the clinical mastitis group and 10.36% in the subclinical mastitis group. The second-largest genus in the clinical mastitis group after antibiotic treatment was *Streptococcus* (12.36%). In the subclinical mastitis group after antibiotic treatment, the second-largest genus was *Corynebacterium* (4.94%). The abundance of *Lactobacillus* in the study groups was distributed as follows: Healthy group 3.46%, clinical mastitis 4.88%, subclinical mastitis 3.68%, clinical mastitis after antibiotic treatment 5.30%, and subclinical mastitis after antibiotic treatment 3.05%. The maximum abundance of *Bifidobacterium* was found in the milk of healthy cows (1.59%). In the clinical mastitis and subclinical mastitis groups after antibiotic treatment, the abundance of *Bifidobacterium* was 1.29% and 1.56%, respectively. The minimum *Bifidobacterium* abundance was recorded in the sick groups (0.83% and 0.81%). Based on the differential analysis of abundance using the DESeq2 package, we could identify differences in the bacterial composition of milk samples from different groups. We performed a pairwise comparison between groups with different forms of mastitis and the healthy group (Figures-2 and 3), which also revealed differences between the clinical and subclinical mastitis groups. Moreover, we analyzed the bacterial composition after the antibiotic treatment of mastitis to determine its effectiveness and impact on the overall microbiome profile (Figures-4 and 5).

A comparative analysis of the microbiome composition in the clinical mastitis group revealed statistically significant differences compared with that in the healthy group. We observed an increase in the abundance of *Hymenobacter* (p = 0.004) and *Lachnospiraceae NK4A136 group* (p = 0.04) in the milk of cows with mastitis. Meanwhile, there was a decrease in the abundance of *Corynebacterium* (p = 0.001), *Atopostipes* (p = 0.02), *Lachnospiraceae NK3A20 group* (p = 0.003), *Facklamia* (p = 0.02), *Aerococcus* (p = 0.02), *Acetitomaculum* (p = 0.03), *Caulobacter* (p = 0.001), *Weissella* (p = 0.03), *Bosea* (p = 0.01), and *Ralstonia* (p = 0.0002) in the mastitis group.

Comparison of the microbiome between the subclinical mastitis group and the healthy group revealed an increase in the abundance of *Hymenobacter* (p = 0.001), *Massilia* (p = 0.01), and *Methylobacterium*–*Methylorubrum* (p = 0.05) and a decrease in the abundance of *Lachnospiraceae NK3A20 group* (p = 0.005), *Caulobacter* (p = 0.04), *Streptococcus* (p = 0.005), and *Ralstonia* (p = 0.005).

When the differences between the two forms of mastitis were analyzed, we found that subclinical mastitis was characterized by an increased abundance of *Streptococcus* (p = 0.003) compared with that in the clinical form of mastitis. After the antibiotic treatment of clinical mastitis, we observed an increase in the abundance of *Histophilus* (p = 0.003), *Psychrobacter* (p = 0.04), and *Streptobacillus* (p = 0.04) and a decrease in the abundance of *Streptococcus* (p = 0.002) and *Staphylococcus* (p = 0.002) compared with that in samples collected before treatment.

**Figure-6:**
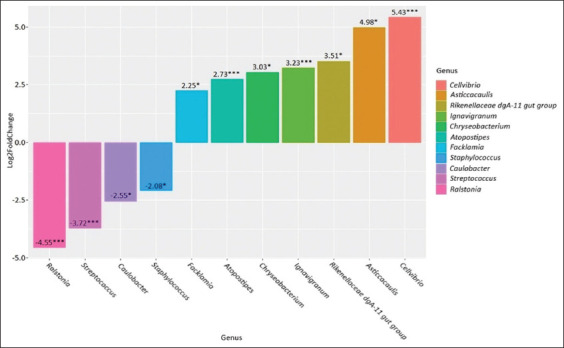
Genus-level differences in the subclinical mastitis group after antibiotic therapy compared to the healthy group. *p ≤ 0.05, **p ≤ 0.01, ***p ≤ 0.001.

**Figure-7:**
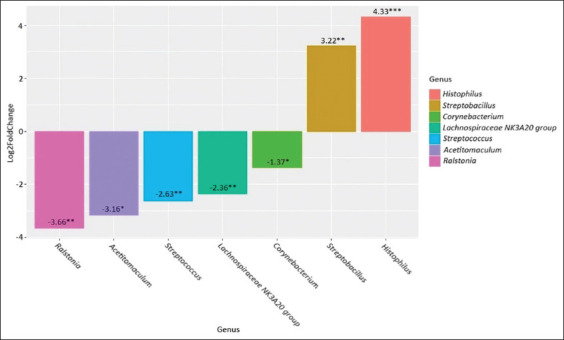
Genus-level differences in the mastitis group after antibiotic therapy compared to the healthy group. *p ≤ 0.05, **p ≤ 0.01, ***p ≤ 0.001.

Regarding the subclinical form of mastitis, after the antibiotic treatment, we observed an increase in the abundance of *Cellvibrio* (p = 4.99E-08), *Asticcacaulis* (p = 0.002), *Ignavigranum* (p = 1.47E-10), *Chryseobacterium* (p = 4.64E-05), *Jeotgalicoccus* (p = 0.001), *Atopostipes* (p = 1.47E-10), *Aerosphaera* (p = 0.04), *Facklamia* (p = 5.76E-06), *Jeotgalibaca* (p = 0.04), *Aerococcus* (p = 0.01), and *Acinetobacter* (p = 0.05), whereas only the abundance of *Staphylococcus* (p = 4.89E-06) showed a decrease. To monitor the recovery of microbiota after the antibiotic treatment, we compared the groups of treated mastitis with the healthy group (Figures-6 and 7).

A differential analysis of differences showed that after antibiotic treatment in the group with subclinical mastitis, there was an increase in the abundance of *Cellvibrio* (p = 4.1E-06), *Asticcacaulis* (p = 0.04), *Rikenellaceae dgA-11 gut group* (p = 0.04), *Ignavigranum* (p = 9.2E-06), *Chryseobacterium* (p = 0.01), *Atopostipes* (p = 9.2E-06), and *Facklamia* (p = 0.01) compared with that in the healthy group. However, we observed a decrease in the abundance of *Staphylococcus* (p = 0.04), *Caulobacter* (p = 0.01), *Streptococcus* (p = 5.5E-06), and *Ralstonia* (p = 0.0001).

**Figure-8:**
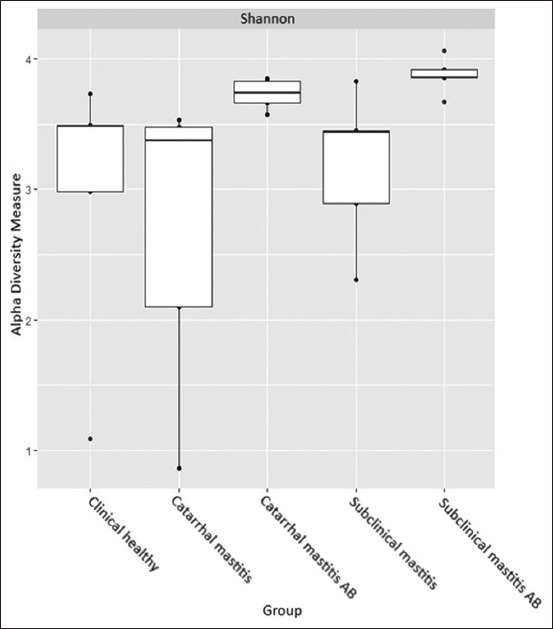
Alpha-diversity measures.

A comparison of the treated clinical mastitis group with the healthy group revealed an increase in the abundance of *Histophilus* (p = 0.0004) and *Streptobacillus* (p = 0.004) and a decrease in the abundance of *Corynebacterium* (p = 0.04), *Lachnospiraceae NK3A20 group* (p = 0.004), *Streptococcus* (p = 0.005), *Acetitomaculum* (p = 0.04), and *Ralstonia* (p = 0.004). In addition to the differential abundance analysis, we analyzed the alpha and beta diversity for the study groups. The Shannon index was used to calculate alpha diversity, and the presence of statistical differences was evaluated using a pairwise test with the Wilcoxon rank sum test, adjusted using the FDR method (Figure-8).

**Figure-9:**
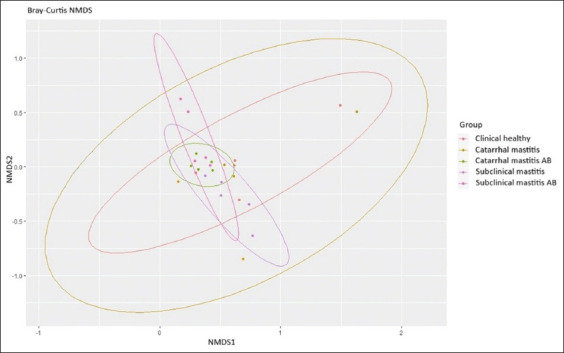
Beta-diversity measures.

We observed the highest alpha diversity for samples collected from the subclinical mastitis group after antibiotic therapy (H = 3.87), and the lowest alpha diversity was typical for samples collected from the clinical mastitis group (H = 2.67). Nonetheless, there were no statistically significant differences in the alpha diversity index between the study groups. We used the Bray-Curtis dissimilarity in combination with NMDS to estimate beta diversity scores (Figure-9) and found no significant differences between the study groups, wherein all of them formed a similar microbiome centroid.

## Discussion

We detected an increase in the abundance of *Hymenobacter* in both types of cow mastitis compared with that in healthy cows. The members of this genus have been previously isolated from various sources, such as mountain soil, sand, fresh water, and sediments [12]. Furthermore, we found several publications devoted to the study of the microbiome of dairy cows that demonstrated an association between *Hymenobacter* and high milk yields and also reported that this genus had previously been identified in the microbiota of the teat canal [13, 14]. Moreover, an increased abundance of bacteria of the genus *Hymenobacter* was detected in the mammary gland tissue of a mouse with clinical mastitis [15]. Our study establishes an association of this genus with the development of the two forms of mastitis in lactating cows.

*Lachnospiraceae NK4A136* has been previously associated with the development of mastitis in cows. Nevertheless, previous research by Zhong *et al*. [16] has shown an increase in the abundance of this taxon in the rumen of cows with mastitis, and not in milk. *Lachnospiraceae NK4A136* has also been demonstrated to be associated with reduced milk production in sheep [17]. Our study also confirmed the possible pathological role of *Lachnospiraceae NK4A136* in the development of clinical mastitis.

Another finding in our study was a statistically significant decrease in the abundance of *Corynebacterium* in the milk samples of cows with clinical mastitis before and after antibiotic treatment compared with that in the healthy group. *Corynebacterium* spp. are commonly detected in milk samples, but it is often unclear whether the cause of their presence is contamination, nonpathogenic colonization, or infection. Previous studies have shown an increase in the abundance of *Corynebacterium* spp. in both subclinical mastitis and clinical mastitis cases [18, 19]. Nonetheless, *Corynebacterium* spp. are also regularly isolated from the milk samples of cows without any signs of mastitis [20]. Furthermore, it is believed that this genus can exert beneficial and antagonistic effects on other pathogenic bacteria [19, 21]. In our study, it was also difficult to determine what role the reduction of *Corynebacterium* abundance in pathological samples may play. We can attribute this problem to the possible greater contamination when collecting healthy samples or the beneficial effect of these bacteria. We also observed an increase in their abundance after antibiotic therapy in both forms of mastitis.

The genus *Atopostipes* was first isolated from pig manure in 2004 [22]. It is a characteristic genus of the porcine gut microbiota and has also previously been identified as a component of the microbiome of the outer udder skin of calves [23, 24]. At present, there is limited knowledge regarding the contribution of *Atopostipes* to cattle health or milk safety. Pang *et al*. [25] found a reduction in the abundance of this genus in a group of cows with mastitis compared with that in healthy cows. Similar findings were observed in a study on the microbiota associated with mastitis in camels [26]. Our results are also consistent with previous data, as we observed a decrease in the abundance of this genus in the clinical mastitis group compared with that in the healthy group. Furthermore, after antibiotic treatment, the milk samples of cows with subclinical mastitis showed an increase in the abundance of *Atopostipes* compared with that in the healthy group and the untreated subclinical mastitis group.

The abundance of *Lachnospiraceae NK3A20 group* was statistically lower in both forms of mastitis, and in the clinical mastitis group after antibiotic treatment, compared with that in the healthy group. This finding may indicate a beneficial role of this genus in the health of the host. However, there are no sufficient studies to describe the effect of *Lachnospiraceae NK3A20 group* on lactating cows. We could only find the study by Martinez Boggio *et al*. [17] that demonstrated that this genus positively correlated with milk yield in sheep.

*Facklamia* is a common bacterial genus found in the teat cisterns and teat canal microbiota of healthy cows [27]. It is one of the commensal genera of cow milk [28]. However, a recent study showed that *Facklamia* abundance was higher in cows with clinically confirmed mastitis than in healthy cows [29]. Our results are most likely consistent with the abundance of this genus being a commensal in cow milk, as we found an elevated abundance in the healthy group compared with the incidence of mastitis, and its abundance was also elevated in the treated subclinical mastitis group compared with that in the untreated and healthy groups.

*Aerococcus* is a genus of environmental bacteria associated with bovine mastitis and other human and animal pathologies [30–32]. However, its exact role in the development of subclinical mastitis and the changes it causes in milk characteristics are not known. It is also often found in barn, air, and dust samples and therefore can be considered a contaminating agent [33]. Despite the large number of studies considering the pathogenic role of *Aerococcus*, in our study, we found an increased abundance in the milk samples of healthy cows compared with that in cows with clinical mastitis. We also observed an increase in the abundance of this genus in the subclinical mastitis group after antibiotic therapy compared with that before antibiotic therapy. Considering the fact that the abundance of this genus in all groups was <1%, in this case, we would not consider *Aerococcus* as a pathogen, but it was most probably a contaminating agent in our study.

The abundance of representatives of the genus *Acetitomaculum* in the group of healthy cows statistically significantly exceeded that in the group of cows with clinical mastitis and subclinical mastitis after antibiotic therapy. This observation is consistent with previous studies by Wang *et al*. [34], Xiang *et al*. [35], wherein one study reported the presence of *Acetitomaculum* among the 25 most common bacterial genera in the rumen of healthy cows, and another study reported a high relative abundance in the rumen, and it was considered to possess the property of producing butyric acid.

Although in most studies, *Caulobacter* is considered to be involved in the development of mastitis in dairy cows [36], in our study, we detected an increased abundance of this genus in the milk samples of healthy cows. A typical member of the *Caulobacter* genus is the aquatic bacterium *Caulobacter leidyia*, whose presence in the milk samples may be due to water contamination during sampling or the large numbers in the parlor, where they may have the opportunity to enter the udder [37].

The genus *Weissella* belongs to the lactic acid bacteria group and is often found in the microbiome of humans and animals. Most species of this genus are commensals of the gastrointestinal tract and are also candidates for probiotics to improve the microbiota and treat various diseases [38]. Despite these positive roles, some strains demonstrate the ability to act as opportunistic pathogens; for instance, strains of *Weissella paramesenteroides* and *Weissella cibaria* have been isolated from the milk of cows with clinical mastitis [39]. A study by Wald *et al*. [40] also indicated a possible association between certain *Weissella* species and the occurrence of intramammary infections in cattle. Nevertheless, most representatives of this genus exert a rather positive effect on the health of the host, which is evidenced by the presence of *Weissella* as a natural colonizer of the gastrointestinal tract of humans and animals, and moreover, these bacteria are widely used as a food product in natural fermentation [39]. In our study, as we observed a decrease in the abundance of representatives of this genus in the clinical mastitis group, we can infer that the microbiota of the investigated milk samples was represented by commensal species, and such an abrupt change in the abundance of these bacteria just indicates a disease state; however, this assumption certainly requires additional confirmation.

Bacteria of the genus *Ralstonia* are often associated with water pollution, including purified water systems [41]. These bacteria are known to be involved in causing various pathological conditions; for instance, the relationship between an increase in the abundance of these bacteria and the development of ulcerative colitis has been demonstrated in humans [42]. However, in our study, the relative abundance of bacteria of the genus *Ralstonia* was considerably reduced in the milk samples of cows with clinical mastitis compared with that in healthy cows. Kuehn *et al*. [43] demonstrated the same change in the abundance of this genus. We found no other references to this bacterium in studies on mastitis. Hence, a change in the abundance of bacteria of the genus *Ralstonia* may serve as a marker of mastitis. However, the antibiotic treatment of cows did not affect the abundance of these bacteria.

The genus *Massilia* consists of gram-negative bacteria of the family *Oxalobacteraceae* [44]. The members of this genus were primarily isolated from environmental sources [45, 46]; however, in rare cases, some species are also known to cause infections in immunocompromised patients [47–49]. The abundance of bacteria of this genus was found to be increased in the intestinal microbiome of patients with viral gastroenteritis [50]. In our study, the abundance of these bacteria increased in the subclinical mastitis group, but no such difference was found in the clinical mastitis group. These bacteria do not play a decisive role in the development of a particular pathology but are opportunistic. We observed the same result with bacteria of the genera *Methylobacterium-Methylorubrum* and *Bosea* because their origin is generally considered ecological [51].

To date, 163 species of *Streptococcus* have been identified, some of which can cause a wide range of infections, including mastitis [52], whereas other species belong to the human commensal microbiota and animals [53]. Our results revealed a significantly less abundance of bacteria of the genus *Streptococcus* in the subclinical mastitis group compared with that in the healthy group, which most possibly indicates a decrease in the abundance of commensal representatives of this genus. Nevertheless, after antibiotic treatment, there was also a decrease in the relative abundance of *Streptococcus* in the clinical mastitis group, which does not allow us to draw unambiguous conclusions regarding these bacteria in our study.

Within the genus *Histophilus*, only one species has been described, namely, *Histophilus somni* [54]. It has been demonstrated that these bacteria are commensal [55] but can also become opportunistic pathogens, causing various clinical signs depending on the affected system [56]. Because of the ambiguous role of this bacterium, it is difficult to evaluate its contribution to the development of mastitis.

After the antibiotic treatment of cows with clinical mastitis, there was an increase in the relative abundance of *Psychrobacter* bacteria compared with that in cows before treatment. *Psychrobacter* is often found in milk [57]. This genus is probably primarily a commensal that decomposes various dissolved organic carbon compounds, except for sugars [58]. In our study, it is not necessary to discuss about the prognostic role of this bacterium; most probably, it was just one of the beneficial effects of antibiotic treatment because there was no difference in the abundance of this bacterium in the clinical or subclinical mastitis group compared with that in the healthy group.

Bacteria of the genus *Streptobacillus* are known to be the most abundant in the vaginal microbiome of cows [59]. However, we could not find any presence of these bacteria in the milk of cows, so it is quite difficult to draw unambiguous conclusions about *Streptobacillus* spp., and further research is necessary.

Bacterial species of the genus *Staphylococcus* are the primary cause of mastitis in cattle worldwide. These bacteria are also capable of exhibiting virulence factors, including, but not limited to, biofilm, surface proteins, exotoxins, membrane-damaging toxins, mutations, and increased antibiotic resistance [60]. Because of this ability, the genus *Staphylococcus* can suspend treatment in dairy enterprises [61]. Pumipuntu *et al*. [62] reported that both molecular characteristics and virulence genes antibiograms of *Staphylococcus* spp. showed an increasing trend of methicillin resistance and multiple antibiotic resistance. *Staphylococcus* has also been reported to be widespread in both healthy milk samples and mastitis milk samples from farms in the central region of Russia [63]. Our study showed a decrease in the abundance of *Staphylococcus* in the clinical mastitis group after antibiotic treatment compared with that before treatment.

Representatives of the bacterial genus *Cellvibrio* are Gram-negative aerobic organisms that can break down cellulose and glucose. This genus has been previously identified on farms that use hay and straw as bedding [64]. Moreover, bacteria belonging to *Cellvibrio* have been cultured from polluted water bodies and found to be lytic of cyanobacteria [64], suggesting the need for the biological control of raw materials used for animal bedding to prevent the development of cattle diseases [65]. In our study, this bacterial genus was detected in the milk samples of cows with subclinical and clinical mastitis after antibiotic treatment compared with that in the control.

Moreover, in our study, we observed an increase in the abundance of *Asticcacaulis* in the subclinical and clinical mastitis groups after antibiotic treatment compared with that in the control, and a previous study by Oikonomou *et al*. [66] also reported that this genus is predominant in the milk of healthy cows.

Bacteria of the genus *Ignavigranum* are also known to cause mastitis and are associated with a subclinical form [67, 68]. This was confirmed in a metagenomics study of milk samples of cows with subclinical mastitis that revealed the composition of the microbiome, in which a predominant bacterial genus was *Ignavigranum* [67]. *Chryseobacterium* is a Gram-negative bacterium that can promote pasteurized milk spoilage and recontamination, reducing milk quality [69]. *Chryseobacterium* has also been found to be an opportunistic bacterium in raw milk. Hagi *et al*. [70] reported the presence of *Chryseobacterium* in the milk of healthy cows, and Kuang *et al*. [71] reported it in the milk of cows with mastitis. An association between this bacterial genus and subclinical mastitis has also been reported [28]. In our study, these bacterial genera were associated with the subclinical mastitis group after antibiotic treatment, wherein there was an increase in the abundance of *Ignavigranum* and *Chryseobacterium* compared with that in the same group before treatment, as well as the control group.

In the subclinical mastitis group, we observed an increase in the abundance of *Aerosphaera* after antibiotic treatment compared with that before treatment; this bacterial genus was previously identified by Kusumawati *et al*. [67] in the milk of cows with subclinical mastitis.

*Acinetobacter* species are aerobic, Gram-negative, non-fermentative bacteria widely distributed in nature, frequently found on teat skin and in cow milk [65, 72]. It can also degrade antibiotics and rapidly develop resistance to them [73]. This genus has previously been reported by Hoque *et al*. [74] to be associated with clinical mastitis in cows. *Acinetobacter* has also been identified not only in the milk of cows with subclinical mastitis but also in the milk of healthy cows [75]. In our study, *Acinetobacter* was identified in the subclinical mastitis group after antibiotic treatment.

Regarding *Jeotgalibaca* and *Rikenellaceae dgA-11 gut group*, there were no published data on correlation with various types of mastitis in cows. However, data have been reported on the association of *Jeotgalibaca* with cattle rumen and manure fermentation [76], as well as its uniqueness for dairy and beef cattle breeding [77]. *Jeotgalibaca* has also been reported to be a major genus identified in milk [78]. Furthermore, *Rikenellaceae dgA-11 gut group* was previously identified by Amat *et al*. [79] in the vaginal microbial community of virgin yearling heifers, and a higher relative abundance of this genus was also observed in the intestines of vaccinated *E. coli* calves than in unvaccinated groups [80]. In contrast to the literature data, our study demonstrated the presence of *Jeotgalibaca* and *Rikenellaceae dgA-11 gut group* in cows with subclinical mastitis after antibiotic treatment.

## Conclusion

Understanding the microbiome of cattle is critical for maintaining their health and productivity. Research in this area can help develop preventive and therapeutic approaches for various diseases, including mastitis, which is one of the most cost-effective infections in the dairy industry. The results of the present study demonstrate a strong correlation between milk microbiome and production efficiency. Our results revealed that several taxa such as *Hymenobacter, Ralstonia, Lachnospiraceae NK4A136*, and *Atopostipes* may be associated with the development of subclinical and clinical mastitis. Bacteria that undergo significant quantitative changes may serve as indicators of subclinical or clinical mastitis.

In addition, our study demonstrated that antibiotics reduced *Streptococcus* levels compared with healthy cows and cows before antibiotic treatment. In addition, antibiotic therapy increased the abundance of beneficial bacteria belonging to the genus *Asticcacaulis*. In conclusion, this study identified a potential link between milk microbiome and the development of mastitis.

## Data Availability

Sequencing data are available in NCBI BioProject database (BioProject: PRJNA736244).

## Authors’ Contributions

VM, VZ, IL, SS, EM, and MS: Conceived the idea, designed the study, developed the theory, and prepared the tools and materials. MG: Collected the data and data analysis. IB, MG, YS, and PM: Analyzed the data and wrote the manuscript with input from all authors. All authors participated in the discussion of the results and contributed to the final manuscript, provided critical feedback, and helped shape the study, analysis, and manuscript. All authors also have read, reviewed, and approved the final manuscript.
